# Economic evaluation of an early childhood development center–based agriculture and nutrition intervention in Malawi

**DOI:** 10.1007/s12571-021-01203-6

**Published:** 2021-08-24

**Authors:** Gelli A, Kemp CG, Margolies A, Twalibu A, Katundu M, Levin C

**Affiliations:** 1 International Food Policy Research Institute, Washington, D.C. USA; 2 Department of Global Health, University of Washington, Washington, USA; 3 Consultant, International Food Policy Research Institute, Washington, D.C. USA; 4 Chancellor College, University of Malawi, Zomba, Malawi

**Keywords:** Cost-effectiveness, Nutrition-sensitive, Africa, Agriculture, Malnutrition

## Abstract

Malnutrition is a leading cause of death and disability among children in low-income countries. Nutrition-sensitive interventions show promise in increasing food access and improving diets. There are possible synergies of integrating these programs with other sectors, improving effectiveness by leveraging resources. However, economic evaluations of these multi-sectoral programs are limited. We aimed to estimate the cost efficiency, cost-effectiveness, benefit-cost ratio, and net benefit of using community-based early childhood development (ECD) centers as platforms for an intervention promoting agricultural production and nutrition among households with young children in Malawi. The intervention was costed using bottom-up micro-costing and top-down expenditure analysis with a societal perspective and a 12-month horizon. Effectiveness estimates were derived from a cluster-randomized control trial. Premature deaths and stunting cases averted were estimated using the Lived Saved Tool. We calculated DALYs averted, and the value of three benefits streams resulting from reductions in premature mortality, increases in lifetime productivity and household agricultural productivity. We transferred the US value of a statistical life (VSL) to Malawi using an income elasticity of 1.5, and a 10% discount rate. Probabilistic sensitivity analysis was conducted using a Monte Carlo model. The intervention cost $197,377, reaching 4,806 beneficiaries at $41 per beneficiary, $595 per case of stunting, $18,310 per death, and $516 per DALY averted. Net benefit estimates ranged from $507,589 to $4,678,258, and benefit-cost ratios from 3.57 to 24.70. Sensitivity analyses confirmed a positive return on investment. Implementing agriculture-nutrition interventions through ECD platforms may be an efficient use of resources in Malawi and similar contexts.

## Introduction

Globally, 250 million children are estimated to be at risk of not reaching their developmental potential due to poverty, malnutrition and other adversities (Black et al., 2013). Poverty, malnutrition and other deprivations during infancy and early childhood can have long-term consequences on cognition and development (Black et al., 2017). Nutrition-sensitive programs, including interventions in early childhood development (ECD), can provide platforms to provide nutrition interventions at scale (Ruel & Alderman, 2013). These programs provide an opportunity for coverage of children outside the priority age group for nutrition interventions (<24 months) and potentially reach caregivers of their younger siblings. Rigorous studies and systematic reviews have shown that ECD interventions can improve children’s cognitive, motor and socio-emotional development (Alderman & Fernald, 2017; Black et al., 2017). Furthermore, ECD interventions are considered to be among the most cost-effective human capital investments (Heckman, 2006). While the evidence on the effectiveness of nutrition sensitive programs and platforms is growing (Ruel et al., 2018), there is little or no evidence on the costs and cost and benefits of multisectoral strategies that combine both nutrition-specific and nutrition-sensitive interventions. Two recent systematic reviews of economic evaluations focus predominantly on preventive and therapeutic nutrition specific interventions, such as micronutrient supplementation, infant and young child feeding, treatment of moderate and severe acute malnutrition, food fortification, with more limited evidence on nutrition sensitive programs, such biofortification and the use of cash transfers to improve nutritional outcomes (Ramponi et al., 2020; Njuguna et al., 2020). Moreover, there are no standardized methods for aggregating the multiple outcomes along the complex pathways linking activities to impacts of nutrition-sensitive strategies (Ramponi et al., 2020).

### Application of economic evaluation methods to multisectoral programs

Economic evaluations can be categorized by what they measure, in terms of costs or resources used and the resulting outcomes or benefits obtained. For all economic evaluations, resources are quantified and typically measured in monetary costs. For health economic evaluations, health improvements can be aggregated over multiple diseases using indicators of mortality, as deaths averted or years of life lost (YLLs), and morbidity, where each year is weighted by the burden of living with each disease, and quantified in aggregate measures such as quality-adjusted life years (QALYs) or disability-adjusted life years (DALYs)(Drummond et al., 2015). Comparing a monetary cost to the YLLs, QALYs or DALYs saved by each intervention can then provide guidance to decision-makers on the most cost-effective intervention to improve health. When there is interest in comparing interventions with multiple benefits or in comparing health improvements to other kinds of gains, such as sustainable agricultural practices or girls’ education, outcomes can be aggregated using monetary units and different interventions can be compared in terms of their benefit-cost ratio. Cost-benefit analysis (CBA) requires monetizing health benefits - that is, placing a dollar value on the number of deaths averted or the life-years gained. While CBA is a popular method for decisions about the advisability of allocating resources to investment projects, until recently it has been less well accepted for evaluating investments in the health sector or other social sectors (Mills, 2014). Placing a dollar value on health benefits has faced both conceptual and empirical difficulties which have recently been addressed through a series of guidance papers developed by Harvard School of Public Health (Robinson et al., 2019).

Ideally, national governments would want to implement the full range of interventions that result in improved health and nutrition. However, given constrained budgetary demands and constraints, economic evaluation is important for setting priorities. Thus, donors, international financial institutions, country-level program designers and policy makers need rigorous cost and cost-effectiveness information to guide strategic planning, financial projections and priority setting. However, there are significant gaps in the evidence on the costs and cost-effectiveness of (a) multisectoral and integrated approaches to improve nutrition, (b) interventions that address the marketing, availability and price of healthy and unhealthy foods, and (c) behavior change communication (BCC) and social marketing to increase demand for nutritious foods and promote healthy behaviors. In addition, multisectoral programs, including agriculture-nutrition interventions, pose additional challenges for economic evaluation. First, given the complex determinants of nutrition outcomes, multiple sectors contribute nutrition improvements and many of the interventions and actions to improve nutritional status have secondary benefits related to food security, production diversity, dietary diversity, women’s empowerment, and higher educational enrollment that are not fully captured in either cost-effectiveness or benefit-cost analyses. Second, there is no current guidance or specific methods on how to value the full range of benefits and opportunity costs incurred by all stakeholders across sectors (Remme et al., 2017; Ramponi et al., 2020). As a result, the interpretation and comparisons of findings from existing economic evaluations in the literature is challenging for policymakers (Ramponi et al., 2020). Given these limitations in current approaches, this study aims to explore whether one economic evaluation method or the other might be preferred, and whether cost-effectiveness and benefit cost analysis approaches provide comparable results yielding the same policy conclusion in terms of value for money. The results of this analysis can help demonstrate the impact of current methodological challenges and inform future approaches for standardized and improved measurement of costs and benefits of multisectoral nutrition strategies. In addition, we describe the methods of the economic evaluation in full detail to allow for ease of interpretation and future potential synthesis.

The primary objective of this study is to apply standard economic evaluation methods to estimate the cost-effectiveness and return on investment (ROI) of a nutrition sensitive intervention scaled-up through an ECD platform in Malawi. The rest of the paper is structured as follows: we first provide an overview of the country context and intervention implemented in Malawi; describe in detail the methods involved in the economic evaluation and the study findings, then discuss the results and limitations, with a focus on the challenges of applying standard economic evaluation methods to complex multisectoral nutrition programs, and conclude.

### Country context

Malawi has one of the highest rates of chronic malnutrition in the world, with 37% of children aged 6-59 months moderately or severely stunted (National Statistical Office, 2017). Malnutrition in rural Malawi is driven by food insecurity, where severe climate-related shocks resulted in approximately 3 million people requiring humanitarian support in 2014-16 (Government of Malawi, 2015; UNICEF, n.d.). Problems of food access and availability, poor feeding practices and diet preferences result in young children in Malawi consuming low-quality diets lacking important micronutrients (Darmon et al., 2002; Ferguson et al., 1993). These micronutrient deficiencies impair children’s physical and mental development (Bailey et al., 2015).

In Malawi, the national ECD program includes preschools (known as Community-Based Childcare Centers (CBCCs)) and parenting groups (Neuman et al., 2014). CBCCs are volunteer-based, community-owned centers that support child development by providing a stimulating and safe environment. The Nutrition Embedded Evaluation Program Impact Evaluation (NEEP-IE) provided rigorous evidence of CBCCs as an effective platform to scale-up nutrition-sensitive interventions in Malawi (Gelli et al., 2017). This intervention provided agricultural inputs and nutrition behavior change communication (BCC) to households, with CBCCs supported by volunteer in-kind and cash contributions. A randomized trial found that the intervention improved food production diversity, maternal knowledge, nutrition practices at the household level and diets of preschoolers and their younger siblings, as well as improving linear growth and reducing the prevalence stunting in younger siblings (Gelli et al., 2018).

### The intervention

A nutrition-sensitive intervention was implemented in Zomba district in Southern Malawi by Save the Children. The intervention aimed to improve the diets and nutrition-related knowledge and care practices in households with infants and young children. The nutrition component of the intervention included behavior change communication (BCC) activities to involve parents and community caregivers in the preparation and planning of meals in the CBCCs, and to promote optimal household feeding and caring practices through parenting groups. Nutrition activities included providing information on topics such as the nutrition needs of infants and young children, food selection and preparation, safety, storage and preservation, meal planning and monitoring as well as hygiene. Recipes included the preparation of nutrient rich meals based on seasonal foods. The agriculture activities focused on improving nutritious food production and on promoting food diversification by using CBCC gardens as demonstration plots. Agricultural support activities included input provision (i.e. ten chicks per household and seeds) and trainings on production of nutritious food (animal source foods, vitamin A rich staples such as orange maize (known as *mthikinya*) and bio-fortified orange–fleshed sweet potato, legumes and nuts, and green leafy vegetables). Agriculture Extension Development Officers visited the community monthly to check progress and address problems. Village savings and loans groups were also formed to encourage communities to generate funds to start small businesses and to contribute to CBCC meal provision and improvement.

In the control group of the randomized trial, communities received training based on standard Government materials, including topics on child nutrition, stimulation and parental roles in school readiness. Caregiver groups were mentored on a monthly basis by trained facilitators for the program’s duration.

#### Program impact pathways

**Fig. 1:**
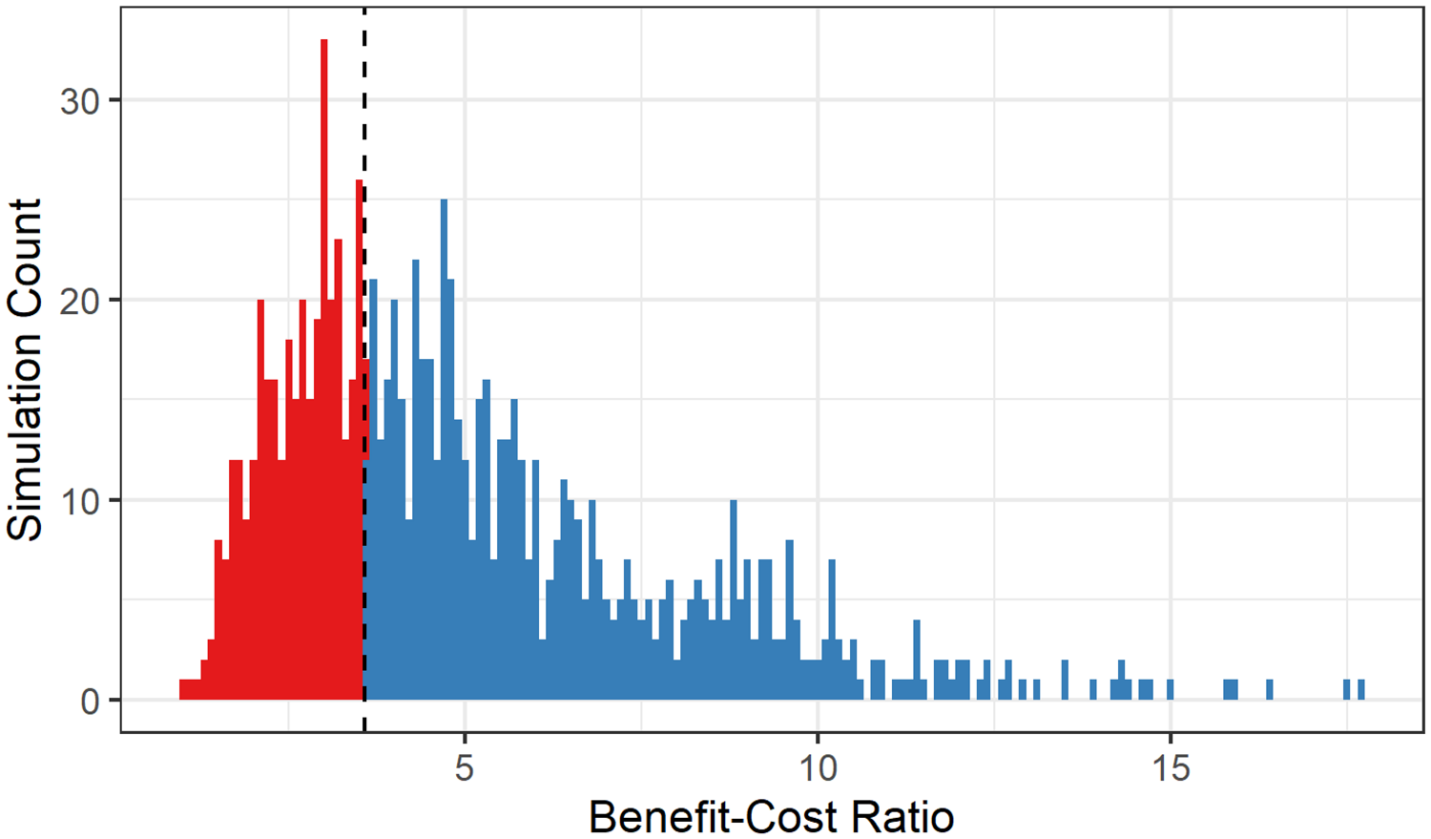
Distribution of benefit-cost ratio estimates from a Monte Carlo simulation

The program theory for the integrated agriculture and nutrition intervention was guided by the Lancet Series framework on Maternal and Child Nutrition (Black et al., 2013) and included three main channels (Fig. 1). First, the intervention could affect agriculture by increasing production, improving the household-level availability of nutritious foods. Second, the nutrition BCC could improve diets and feeding practices by improving caregiver knowledge. And third, by increasing the regularity and quality of CBCC meals, the intervention could influence CBCC participation, possibly enhancing both child learning and nutritional status.

## Methodology

This study was guided by the economic evaluation framework developed as part of the Strengthening Economic Evaluation for Multisectoral Nutrition Strategies (SEEMS) initiative funded by the Bill and Melinda Gates Foundation and led by the University of Washington in collaboration with the International Food Policy Research Institute. The SEEMS-Nutrition approach standardized both cost analysis, including data collection, cost coding, allocation, integration of costs and the final cost analysis, and for benefit valuation across complex multisectoral (early childhood development, agriculture and nutrition) program impact pathways (Levin et al., 2019). The benefit-cost analysis was conducted in alignment with the Reference Case Guidelines for Benefit-Cost Analysis in Global Health and Development (Robinson et al., 2019).

### Costs

The cost analysis estimated the incremental economic costs of providing an integrated agriculture and nutrition intervention through an ECD platform from a societal perspective. The detailed methods for the cost analysis are published elsewhere (Margolies et al., 2021). Briefly, a mixed methods approach was used to measure and allocate costs for program activities and inputs using expenditure data combined with micro-costing to capture all resource inputs and economic costs. All costs were allocated to a set of standard input and cost categories for multisectoral strategies to improve nutrition and health. Major activities over the program lifetime (1y) were listed and categorized as either start-up or recurrent, providing the basis to capture the quantities and costs for all inputs. Start-up activities included the training of trainers and the creation of training materials for promoting the production and consumption of nutritious foods. Community trainings on agricultural production, food processing and nutrition, parenting practices and village savings and loans were among the recurrent activities. Other recurrent activities at the household level included trainings on rearing livestock as well as the provision of in-kind agricultural inputs such as vegetable seeds. Input categories included fixed costs, such as construction materials or capital equipment. Variable costs included personnel, agriculture production supplies, food provision, and consumable cooking supplies.

#### Cost data collection

Financial expenditure data from the 12 months of program implementation were collected from program-level Save the Children administrative records. Expenditure data included the salaries of program staff, frontline workers who provided extension and nutrition support and Government workers, as well as costs spent by the NGO on agricultural inputs, training and other supplies (including vehicle use and fuel). Program staff were interviewed to estimate the allocation of their salaried time to program activities during the implementation period.

Additional information on time allocation and out of pocket expenditures related to CBCC activities were collected retrospectively using semi-structured questionnaires during an in-depth facility survey in all 30 CBCCs covered by the intervention. CBCC cost questions detailed contributions made to individual CBCCs, including in which form they were made. For example, the surveys documented total in-kind (value in MKW) or financial costs (cash in MKW) of contributions, the frequency of contribution, who made the contribution and then probed on each category of CBCC engagement explained by those involved in the intervention (i.e. construction, maintenance, condiments, food, fuel, building supplies, roofing).

Program-related beneficiary opportunity costs were estimated by collecting time allocation data through the household survey (Margolies, 2019). The time allocation module was adapted from the Women’s Empowerment in Agriculture Index (WEAI) (Alkire et al., 2013) and updated to include program activities. The questionnaires captured the opportunity costs of beneficiaries to feed into the overall cost analysis. Volunteer labor generally took the form of women helping with childcare and meal preparation in the CBCCs, maintaining gardens and participating on CBCC committees which manage preschool activities. Men in the intervention communities helped construct and maintain CBCC structures or joined CBCC committee activities. Communities donated food in-kind from their own gardens to CBCCs or contributed purchased food. Price data for the food items used in the preschool meals were collected using standard market-level surveys in the four primary markets in the study area. As the study captured incremental costs to the base ECD program, additional costs were not collected at control CBCCs.

#### Cost data analysis

Program activities and expenditures were coded to standardized cost categories, permitting breakdown of the cost drivers by program activity and input. These categories were created as part of the SEEMS-Nutrition methodology for economic evaluations of multisectoral nutrition programs^1^. The SEEMS common approach integrates existing methods and is informed by existing guidelines for economic evaluations in health and agriculture such as the GHCC reference case on global health costing (Vassall et al. 2017) for standardized cost and input categories, among others. The common approach provides the basis for benchmarks for future cost comparisons with other interventions. The opportunity cost of program participation was calculated by multiplying voluntary beneficiary labor time per child per year by 50% of the minimum wage for a 19-year old apprentice (unskilled) worker in Malawi, which is 23 USD per month, as actual wages in Malawi are reported to be less than half of legal wages (Danish Trade Council for International Development and Cooperation, 2014). Beneficiary time contributions were allocated 50% to establishing and running community groups and 50% to school meal preparation. The annual cost per child was estimated by aggregating the average daily cost per child per day which was calculated from individual cost items per child per day from each CBCC. The assumed useful life of capital equipment in preschools was 10 years, with the exception of the agricultural production manual, set to 5 years. Capital costs were annuitized with a discount rate of 3% according to World Bank recommendations. Microsoft Excel was used to code and analyze cost data.

All study participants gave prior informed written or verbal consent. Ethical approval for trial data collection was obtained from the Malawi National Commission for Science and Technology (approved 12/11/2015, ref: NCST/RTT/2/6) and from the International Food Policy Research Institute IRB in Washington, D.C. (approved 26 March 2015, ref: IRB00007490).

### Intervention effectiveness

The details of the NEEP-IE intervention, randomized design and trial findings have been published elsewhere (Gelli et al., 2017, 2018, 2019). Briefly, a longitudinal cluster randomized control trial was implemented in 60 community-based childcare centers (CBCCs) in Zomba district in southern Malawi, covering 1,248 preschool children (aged 36–72mo) and 304 younger siblings (6–24mo). CBCCs were randomized to 1) control group providing Save the Children’s ECD program; or 2) treatment group providing standard ECD program with additional activities to improve nutritious food production and behavior change communication (BCC) to improve diets and care practices for young children. Primary outcomes were household production and production diversity, preschooler enrollment and attendance, and dietary intake measured by quantitative 24-h recall and minimum diet diversity for younger siblings. Secondary outcomes included anthropometry for preschoolers and younger siblings, child development scores for preschoolers, and women’s asset ownership and time use. The timeline for the trial included surveys at baseline (December 2015), mid-line (April 2016) and endline (December 2016).

### Cost-efficiency analysis

Numbers of index preschool children, total beneficiaries (including mothers and younger siblings of index preschool children), and households reached by the intervention were tallied by program staff. Ratios of cost per child, beneficiary, and household reached were calculated by dividing the total estimated intervention cost by the respective numbers reached.

### Cost-effectiveness analysis

The key assumptions and parameters for the cost-effectiveness and benefit-cost analyses are listed in Table 1.

**Table 1:** Key assumptions and parameters for the cost-effectiveness and benefit-cost analyses

	Assumption	Parameter	Source
CEA	Stunting and Premature Mortality Averted		
	Population of implementation area	118,261	(Walker et al., 2013); based on population estimates from (Gelli et al., 2018)
	Proportion of population aged 6-24 months	3.27%	(Walker et al., 2013); based on population estimates from (Gelli et al., 2018)
	DALYs Averted		
	Standard life expectancy at age 1-4	Men: 77.8 years	(Coale et al., 1983)
		Women: 80.3 years	
	Malawi life expectancy at age 1-4	Men: 63.3 years	WHO Global Health Data Observatory (https://apps.who.int/gho/data/view.main.60980)
		Women: 68.5 years	
	Stunting disability weight	0.002	(WHO, 2004)
BCA	Discount Rate	3%	(Wilkinson et al., 2016)
		5%	(Hoddinott et al., 2013a, b; Horton & Hoddinott, 2014; Wong et al., 2019)
		10%	(World Bank)
	Benefits from Avoided Premature Mortality		
	US VSL (2016)	$9,400,000	(Robinson et al., 2019).
	US GNP per capita purchasing power parity (PPP) (2016)	$57,900	(Robinson et al., 2019)
	Malawi GNP per capita PPP (2016)	$1,220	World Bank (https://data.worldbank.org/indicator/NY.GNP.PCAP.PP.CD?locations=MW)
	Malawi adult life expectancy, undiscounted	33.8 years	(WHO Global Health Data Observatory (https://apps.who.int/gho/data/view.main.60980)
	Malawi YLLs due to death at age 2	63.3 years	(WHO Global Health Data Observatory (https://apps.who.int/gho/data/view.main.60980)
	Benefits from Increased Lifetime Productivity		
	Increased wages due to stunting aversion	30%	(Aryeetey et al., 2020)
	Ages of employment	16 to 60 years	
	Average adult wage in Malawi (2016)	$750	(World Bank, 2018)
	Projected Malawi GNI per capita growth %	4.46%	SSSP-IIASA (Riahi et al., 2017)
	Overall prevalence of stunting	37%	(National Statistical Office, 2017)
	Benefits from Increased Household Agricultural Production		
	Years of increased household production	20 years	
	Projected Malawi GNI per capita growth %	4.46%	SSSP-IIASA (Riahi et al., 2017)
	OFSP price/kg (USD)	$0.21	CIP http://www.sweetpotatoknowledge.org/wp-content/uploads/2016/03/PRES14-VANVUGT-VALUE-CHAIN-MALAWI-20160316.pdf
	Brown bean market price/kg (USD)	$0.70	http://afjare.org/wp-content/uploads/2018/01/2.-Dzanja-et-al.pdf
	Pigeon pea market price/kg (USD)	$0.42	
	Groundnut market price/kg (USD)	$0.81	
	Soya bean market price/kg (USD)	$0.42	
	Chicken market price/each (USD)	$7.50	http://www.lrrd.org/lrrd16/12/gaus16097.htm
	Egg market price/each (USD)	$0.15	https://knoema.com/FAOPS2017DEC/fao-producer-price-statistics?country=1000980-malawi
	% of additional production sold at market	50%	

#### Stunting averted

We estimated the total number of stunting cases averted among younger siblings of index preschool children in the implementation area by 1) calculating the number of stunting cases averted within the intervention arm of the trial and 2) scaling that number across an estimated number of children aged 6-24 months in the trial implementation area. We estimated the number of children aged 6-24 months in the implementation area by multiplying the estimated total population of that area (118,261) with the estimated proportion of Malawi’s population aged 6-24 months (3.18%). We then calculated the ratio of the estimated implementation area population aged 6-24 months to the trial sample of children that age and multiplied that ratio by the number of stunting cases averted in the trial population.

#### Premature mortality averted

We estimated the total number of premature deaths averted among younger siblings of index preschool children in the implementation area using the Lives Saved Tool (LiST). Specifically, we used LiST to estimate the number of deaths that would be averted in the implementation area given the relative change in stunting prevalence observed in this trial comparing the intervention and control populations of younger siblings of index preschool children. LiST uses published estimates of the association between suboptimal growth in children and all-cause mortality to model the effects of stunting reduction on child mortality in specific areas, conditional on the current child mortality rate (Olofin et al., 2013).

#### DALYs averted

We estimated Disability-Adjusted Life Years (DALYs) averted due to the reduction in stunting prevalence and subsequent reduction in premature mortality. Years of life lost (YLLs) were calculated assuming that premature mortality due to stunting occurred on average at age 2.6, using standard life expectancies at that age (77.8 for men, 80.3 for women) (Coale et al., 1983). Years lived with disability (YLDs) were calculated assuming non-fatal stunting persisted for the same standard life expectancies, with a disability weight of 0.002 (WHO, 2004). As a sensitivity analysis, we estimated DALYs using Malawi-specific life expectancies (World Health Organization, n.d.). For each of the above, incremental cost-effectiveness ratios (ICERs) were calculated by dividing the incremental cost estimate by the incremental effectiveness estimates.

### Benefit-cost analysis

#### Common assumptions

We used three different discount rates in our benefit estimation: 3%, 5%, and 10%. Three percent follows the recommendations of the iDSI reference case (Wilkinson et al., 2016). We used 5% to be comparable to similar recent benefit-cost analyses (Horton & Hoddinott, 2014; Wong et al., 2019), and we calculated the 10% discount rate by subtracting the inflation rate from the treasury bond rate (World Bank 2018).

As the impact estimates were obtained from an intent-to-treat analysis, a key assumption in terms of the benefits estimation was that households and preschool children in the implementation area who were not enrolled in the trial would respond to the intervention as households and children who enrolled in the trial.

#### Benefits from avoided premature mortality

We valued the estimated reduction in the risk of premature mortality using three alternative population-average approaches, as proposed by Robinson et al. (2019) for settings lacking empirical estimates of willingness to pay for mortality risk reductions. These approaches include: 1) extrapolating the value of a statistical life (VSL) from the US value (USD 9.4m in 2016) with an income elasticity of 1.5, 2) estimating a constant value of statistical life year (VSLY) by dividing the population-average VSL for each year from the first by the undiscounted life expectancy of the average Malawian adult (33.8 years), 3) 160 times the GNI per capita PPP and an income elasticity of 1.0 (US ratio), and 4) 100 times the GNI per capita PPP and an income elasticity of 1.0 (OECD ratio). Income elasticity represents the degree of change in the VSL associated with a change in income; an income elasticity greater than one implies that the ratio of VSL to GNI per capita is smaller in poorer populations than in higher-income settings. Table 2 below presents the results of the VSL estimation.

**Table 2:** Alternative value of a statistical life (VSL) estimates in Malawi

	US VSL Extrapolation	VSLY (Age/Life Expectancy Adjusted)	US Ratio	OECD Ratio
Income elasticity	1.5	1.5	1	1
VSL (Malawi, 2016 International Dollars)	$28,751	$53,844	$195,200	$122,000
VSL (Malawi, 2016 USD Dollars)	$7,389	$13,838	$50,166	$31,354

#### Benefits from increased lifetime productivity

We also valued the long-term benefits of reduced stunting in terms of increased lifetime productivity. We assumed that non-stunted individuals earn on average 30% higher wages in adulthood than stunted individuals and that these increased wages will be earned from age 16 to 60 (Aryeetey et al., 2020; Hoddinott et al., 2013b; Horton & Hoddinott, 2014). We used the average adult wage rate in Malawi in 2016 ($750) (World Bank, 2018), the estimated proportion of Malawians who are stunted (37%) (National Statistical Office, 2017), and the three different discount rates to estimate incremental gains in lifetime wages for individuals with averted stunting in the implementation area, adjusting for the projected GNI per capita growth rate from 2015-2075. To estimate this projected GNI per capita growth rate, we took the average of projected growth estimates over 2015-2075 from the Shared Socioeconomic Pathways Public Database, across all five of their model scenarios (Riahi et al., 2017). To avoid double counting of productivity benefits, deaths averted via stunting reduction were not included in this component of the analysis.

#### Benefits from increased household agricultural production

Finally, we valued the long-term benefits of increased agricultural productivity at the household level in terms of the value of supplementary production of various crops promoted by the program (including orange-fleshed sweet potato, brown bean, groundnut, chicken and eggs). We assumed that households experienced this supplemental production over twenty years, that 50% of supplemental production was sold at market prices, and that market prices increased each year in line with the projected GNI per capita growth rate. Market prices in 2016 were derived from the literature (Table 1: Assumptions). We used three different discount rates to estimate total incremental gains in household income due to the supplemental production of each crop by the households in the implementation area.

Net benefits were calculated by subtracting the estimated incremental intervention cost from the total valued benefit estimates. Benefit-cost ratios were calculated by dividing the total valued benefit estimates by the incremental cost estimate.

### Sensitivity analyses

Probabilistic sensitivity analysis was also performed using a Monte Carlo simulation with 1,000 draws. In this analysis, we varied multiple parameters in the model including the estimated effects on stunting and mortality, the productivity gains from reduced stunting, the projected GNI per capita growth, the proportion of additional agricultural production sold, and the intervention costs. The parameters used in this analysis are documented in Table 3. We did not vary the VSL, as the VSL used in our base case was already the smallest of those we estimated; including larger VSL estimates in the Monte Carlo simulation could only inflate our net benefit and benefit-cost ratio estimates. Parameters for numbers of stunting cases and deaths averted were assumed to be drawn from normal distributions. For both parameters, the mean was assumed to be the base case parameter estimate. Standard deviations for each were calculated from the parameter uncertainty intervals (e.g., the 95% confidence interval for number of deaths averted obtained from LiST). These parameters were also assumed to be correlated (r=0.55). All other parameters were assumed to be drawn from uniform distributions. Productivity gain estimates extended the full range of estimates as documented by previous studies (Guh et al., 2008; Hoddinott et al., 2008); projected GNI per capita growth rates spanned from 2% to 7%, and intervention costs ranged +/- 20% of the base case. Deterministic one-way sensitivity analyses were also performed for all parameters. Sensitivity analyses were performed using Microsoft Excel and Oracle Crystal Ball.

**Table 3:** Monte Carlo simulation parameters

Parameter		
Normal Distribution	Mean	SD
Stunting cases averted	332	82
Deaths averted	11	1
Uniform Distribution	Minimum	Maximum
Increased wages due to stunting aversion	11%	82%
GNI per capita growth %	2%	7%
% of additional production sold at market	25%	75%
Intervention cost	$157,902	$236,853

## Results

The estimates from the effectiveness trial are as follows: younger siblings of index preschool children in the intervention group had greater increases in height-for-age z scores than did children in the control group (DID: 0.44; P < 0.05) and greater reductions in the prevalence of stunting (DID: –17 percentage points; P < 0.05). Household production of nutritious foods increased, including production of biofortified orange-fleshed sweet potato, groundnuts, pigeon peas, soya, chickens, and eggs. See (Gelli et al., 2018) for detailed results.

### Economic analysis

Table 4 presents the results of the economic analysis, including estimates for the costs, cost-efficiency, cost-effectiveness and benefit-cost analyses.

**Table 4:** Economic analysis results

Costs			
Program	$147,916		
Community contribution	$49,461		
Total	$197,377		
Cost-Efficiency			
Preschool children	1,017	$194	$/child
Total beneficiaries	4,806	$41	$/beneficiary
Total households	900	$219	$/household
Cost-Effectiveness			
Stunting cases averted	332	$595	$/stunting case averted
Deaths averted	11	$18,310	$/death averted
DALYs averted (standard LE)	382	$516	$/DALY averted
DALYS averted (Malawi LE)	363	$543	$/DALY averted
Benefit-Cost			
Benefits (US VSL Extrapolation, USD$2016)	3% Discount	5% Discount	10% Discount
Deaths averted	$79,653	$79,653	$79,653
Lifetime productivity	$4,616,473	$2,276,826	$528,044
Agricultural production	$179,509	$147,636	$97,270
Total	$4,875,635	$2,504,115	$704,967
Net benefits	$4,678,258	$2,306,737	**$507,589**
Benefit-cost ratio	24.70	12.69	**3.57**

Bold denotes base case

*LE* life expectancy, *DALY* disability-adjusted life year, *MPA* mean probability of adequacy

#### Costs

The intervention was estimated to cost US$197,377.

#### Cost-efficiency

The intervention served a total of 1,017 index preschool children, 900 households, and 4,806 beneficiaries. The intervention cost was $194 per index preschool child, $219 per household, and $41 per beneficiary reached.

#### Cost-effectiveness

The intervention averted an estimated 332 cases of stunting and 11 premature deaths in the intervention area, resulting in 382 DALYs averted assuming a standard life expectancy at age 1-4y, and 363 DALYs averted assuming the Malawian life expectancy at age 1-4y. The intervention cost $595 per case of stunting averted, $18,310 per death averted, $516 per DALY averted using the standard life expectancy, and $543 per DALY averted using the Malawi life expectancy.

#### Benefit-cost

Estimated total benefits due to aversion of premature mortality ranged from $79,653 using the VSL extrapolated from the US to $540,796 using the VSL equal to 160 times Malawi’s GNP per capita. We used the US-VSL extrapolation for all net benefit and benefit-cost ratio estimates below. Estimated total benefits due to increased lifetime productivity ranged from $528,044 with a 10% discount rate to $4,616,473 with a 3% discount rate. Estimated total benefits due to increased agricultural production ranged from $97,270 with a 10% discount rate to $179,509 with a 3% discount rate. Using the US VSL extrapolation, net benefit estimates ranged from $507,589 to $4,678,258 (base case= $507,589), and benefit-cost ratios ranged from 3.57 to 24.70 (base case= 3.67).

#### Sensitivity analyses

The probabilistic sensitivity analysis results found that 99.9% of the Monte Carlo simulations projected a positive net benefit and a benefit-cost ratio greater than one. The simulated benefit-cost ratios ranged from 0.98 to 17.68 with a mean of 5.12 and a median of 4.45. Figure 1 presents the distribution of benefit-cost ratio estimates; 64.5% of simulations projected a benefit-cost ratio greater than the base case of 3.57.

**Fig. 2:**
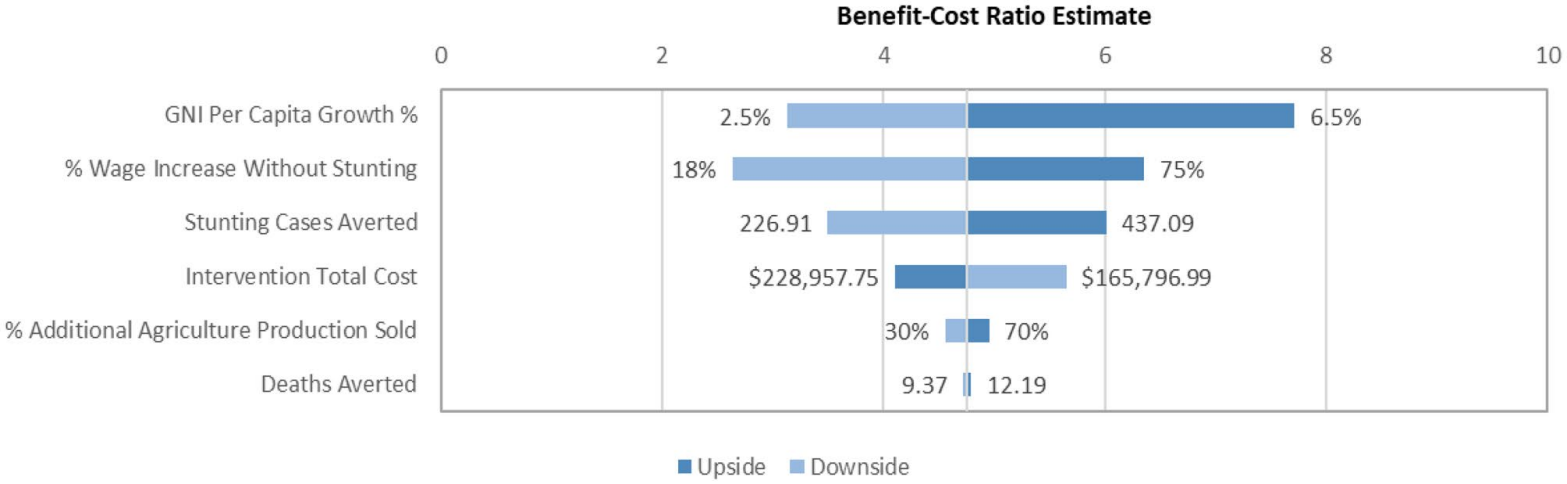
Distribution of benefit-cost ratio estimates from a deterministic one-way sensitivity analysis

Figure 2 displays the results of the deterministic one-way sensitivity analysis. Benefit-cost ratio estimates were most sensitive to changes in the projected GNI per capita growth rate and changes in the wage increase due to stunting aversion. Estimates were least sensitive to changes in the percent of additional agricultural production sold at market prices and changes in numbers of deaths averted.

## Discussion

This study is the first to our knowledge to assess the economic case for using community-based ECD centers as platforms to promote agricultural production, diet, and nutrition in Malawi and similar low-income, high-need contexts. We estimated the cost of the intervention per DALY averted to be slightly higher than Malawi’s per capita GDP, indicating that the intervention is approximately cost-effective in this setting (Robinson et al., 2017). Our benefit-cost analysis suggested that this approach may be an efficient use of resources given the link between stunting reduction and subsequent increased economic productivity and averted premature mortality. The estimates from this study are also in the upper range of similar interventions that are considered cost-effective in the literature (Table 5). Detailed comparisons with estimates from the literature are however difficult to interpret because of the different methodologies employed in collecting and analysing the cost data, obtaining estimates on the impact of interventions and valuing the benefits streams of the intervention impacts (Ramponi et al., 2020). In this study, the two economic evaluation methods provide comparable evidence that this intervention is good value for money, and has a favorable return on investment. Importantly, however, if the trial had not detected an effect of the intervention on the prevalence of stunting among young children in the implementation area, intervention costs would have outweighed the estimated benefits. This particular finding highlights the limitations of applying current economic evaluation approaches to integrated agriculture and nutrition interventions. By design, the objectives of the intervention included improving diet and production diversity, focusing on improving caregiver nutrition knowledge and promotion of a bundle of nutritious foods. The NEEP-IE trial results found evidence of substantive improvements in the dietary intake (including intake of micronutrients including iron and vitamin A) of preschoolers, which were the primary outcomes of the trial. These particular effects were not valued in the economic evaluation and as such don’t factor in the estimation of the economic benefits. The benefits from the intervention we estimated in this analysis were mostly accrued from the benefit streams arising from the effects of the intervention on the younger siblings of the preschoolers involved. This finding highlights a gap in applying the current methodology for the economic evaluation of nutrition specific interventions to multisectoral nutrition-sensitive programs like the one considered in this study. An improved economic evaluation framework is required that includes in its estimates of cost-effectiveness the full breadth of target groups and indicators on the program impact pathways for agriculture and nutrition interventions. Comparisons on the cost-effectiveness of agriculture and nutrition interventions that do not value these effects are underestimating important benefits and opportunity costs and may not be very meaningful nor helpful for policy-makers looking to prioritize multi-sectoral investment decisions. One explicit step requires appropriate valuation of benefits arising from indicators that are more proximal to the intervention activities than indicators of nutrition status or income generation, for example, indicators related to dietary intake, like dietary diversity. The evidence on the positive effects of agriculture and nutrition interventions on this particular indicator is fairly well established (Sharma et al., 2021). Nevertheless, in this particular case, given that the intervention did reduce the prevalence of stunting, albeit not in the primary reference age group for the trial, this study suggests that the approach of integrating nutrition-sensitive agriculture promotion into community-based ECD centers may be as economically efficient as other proven nutrition-specific interventions.

**Table 5:** Cost-effectiveness comparisons to similar interventions

Intervention	Country	Sectors	BCR	Source
Essential nutrition-specific interventions	17 countries	Nutrition, health	18 (3.6 – 48)	Hoddinott et al., 2013a, b
Essential nutrition-specific interventions	Haiti	Nutrition, health	5.2 (2 – 8.4)	Wong & Radin, 2019
School feeding	Nepal	Nutrition, education	5.2 (3.1 – 8.6)	WFP & MasterCard, 2018
*NEEP (Integrated nutrition/ECD)*	*Malawi*	*Nutrition, agriculture*	*3.6 (3.6-24.7)*	Gelli et al., 2019
Rural sanitation project	India	WASH	2.5 – 5	Weiss et al., 2018
Community-led total sanitation	Hypothetical SSA	WASH	1.6 (1.2 – 2)	Radin et al., 2020
Integrated nutrition and ECD	Nicaragua	Nutrition, education	1.5 (1.3-2.3)	Boo et al., 2014

### Strengths and limitations

This study had several key strengths, including drawing on results from the RCT for the estimates of effect sizes and on a full-economic cost analysis of the intervention. These characteristics make this the first study to our knowledge to estimate the cost-effectiveness of a multi-sectoral intervention based on empirical data. Additionally, another strength of this study is that it draws on a broad and comprehensive cost analysis based on diverse data sources such as trial data, community-level surveys, staff interviews and detailed line-item program expenditures. There were also some limitations. First, the estimates on the costs and effects of the NEEP program originate from a relatively small-scale intervention operating in 60 villages in a single district in southern Malawi. In terms of the context-specificity of these estimates, we find per-child estimates for community costs compare relatively well to similar contexts. As such, the results of the trial may provide upper bound for effectiveness if we consider that scale-up of the program is likely to face challenges that could result in sub-optimal implementation fidelity. On the other hand, the costs and cost-structure of the intervention activities as implemented by the NGO are also likely to provide upper bounds, as operating the program at scale is likely to benefit from economies of scale. How these two factors influence the cost-effectiveness of the program at scale remains an important question for future research. Second, though our benefit-cost analysis incorporated and valued a broad range of benefits related to premature mortality averted, increased lifetime economic productivity, and increased household agricultural productivity, some potential benefits were excluded because they were not measured in the trial or because they could not be appropriately valued. For example, the intervention changed gender-based power dynamics at the household level through shifts in women’s asset ownership. Recent evidence suggests that empowering women is not only an important intrinsic benefit of these types of programs but also an important pathway to improving maternal and child nutrition outcomes (Heckert et al., 2019). Lastly, to address any uncertainty around cost estimates such as those due to potential recall bias, we conducted extensive sensitivity analyses to understand potential upper and lower bounds given these uncertanties.

In summary, this study provides rigorous evidence on the return of investment of integrated agriculture and nutrition programs as well as insights on current methodological limitations of economic evaluations in the literature as applied to integrated agriculture and nutrition programs. The evidence presented in this study suggests that integrated nutrition and agriculture interventions implemented through ECD platforms may be an efficient use of financial resources in Malawi and similar contexts. However, further research is needed to address the important gaps that remain on appropriate methods for economic evaluation to allow meaningful interpretation of the evidence on comparisons of cost-effectiveness of multi-sectoral interventions.

## Footnotes

1 Strengthening Economic Evaluation for Multisectoral Strategies for Nutrition (SEEMS-Nutrition). https://sites.google.com/uw.edu/seems-nutrition/home?authuser=0.
